# Chemical Vapor Deposition of Monolayer Mo_1−x_W_x_S_2_ Crystals with Tunable Band Gaps

**DOI:** 10.1038/srep21536

**Published:** 2016-02-22

**Authors:** Ziqian Wang, Pan Liu, Yoshikazu Ito, Shoucong Ning, Yongwen Tan, Takeshi Fujita, Akihiko Hirata, Mingwei Chen

**Affiliations:** 1 Department of Materials Science, Graduate School of Engineering, Tohoku University, Sendai 980-8577, Japan; 2 WPI Advanced Institute for Materials Research, Tohoku University, Sendai 980-8577, Japan; 3 CREST, JST, 4-1-8 Honcho Kawaguchi, Saitama 332-0012, Japan; 4 Department of Mechanical and Aerospace Engineering, School of Engineering, Hong Kong University of Science and Technology, Clear Water Bay, Kowloon, Hong Kong SAR

## Abstract

Band gap engineering of monolayer transition metal dichalcogenides, such as MoS_2_ and WS_2_, is essential for the applications of the two-dimensional (2D) crystals in electronic and optoelectronic devices. Although it is known that chemical mixture can evidently change the band gaps of alloyed Mo_1−x_W_x_S_2_ crystals, the successful growth of Mo_1−x_W_x_S_2_ monolayers with tunable Mo/W ratios has not been realized by conventional chemical vapor deposition. Herein, we developed a low-pressure chemical vapor deposition (LP-CVD) method to grow monolayer Mo_1−x_W_x_S_2_ (x = 0–1) 2D crystals with a wide range of Mo/W ratios. Raman spectroscopy and high-resolution transmission electron microscopy demonstrate the homogeneous mixture of Mo and W in the 2D alloys. Photoluminescence measurements show that the optical band gaps of the monolayer Mo_1−x_W_x_S_2_ crystals strongly depend on the Mo/W ratios and continuously tunable band gap can be achieved by controlling the W or Mo portion by the LP-CVD.

Band gap engineering is indispensable in nowadays electronics and optoelectronics since a variety of artificial band gaps build on the basics for the optimization and innovation in diverse device applications. Recently, the emergence of two dimensional (2D) materials with a direct band gap opens up a new avenue for improving the integrity in devices. Similar to the bulk semiconductors, the band gaps of 2D materials are changeable and can be modified by applying strains^1^,^2^,^3^,^4^,^5^, functionalization^6^,^7^ and chemical doping^8^,^9^,^10^,^11^,^12^,^13^,^14^,^15^,^16^,^17^. As a promising 2D alloy system, the direct band gaps of monolayer Mo_1−x_W_x_S_2_ crystals have been demonstrated to strongly depend on Mo/W ratios^12^,^15^,^18^. Although monolayer Mo_1−x_W_x_S_2_ nanosheets have been obtained by mechanical exfoliation^15^,^19^, it is technically inappropriate to implement the nano-sized fragments into devices for practical applications. On the other hand, while chemical vapor deposition (CVD) has been widely used to grow monolayer MoS_2_ and WS_2_ with high crystallinity^20^,^21^,^22^,^23^,^24^,^25^,^26^,^27^,^28^,^29^,^30^,^31^,^32^,^33^,^34^,^35^,^36^, the atomic-scale uniformly mixed Mo_1−x_W_x_S_2_ 2D alloys have not been successfully achieved by conventional CVD despite many efforts have been devoted to this problem^37^,^38^,^39^. In previous CVD growth of pristine MoS_2_ and WS_2_ monolayers, MoO_3_ and WO_3_ powders have been used as the transition metal sources and S powder as the reductant source^20^,^21^,^22^,^23^,^24^,^25^,^26^,^28^,^29^,^31^,^32^,^33^,^34^,^36^. MoO_3_ is partially reduced by sulfur vapor to form volatile MoO_3−x_ at ~500 °C, and the growth of MoS_2_ can be attained at a temperature as low as 530 °C^21^,^24^. In contrast, WO_3_ or WO_3−x_ has a poor volatility and requires a high temperature up to ~1000 °C for vaporization. Thus, the CVD temperature for WS_2_ growth is not lower than 800 °C by using WO_3_ or WO_3−x_ as the precursor^26^,^28^,^33^. The large CVD temperature gap between MoS_2_ and WS_2_ growth makes it difficult to control the supply of the two oxide precursors for the synthesis of monolayer Mo_1−x_W_x_S_2_ alloys. As a result, MoS_2_ is usually deposited prior to WS_2_, resulting in the formation of two-phase MoS_2_/WS_2_ heterostructures, rather than Mo_1−x_W_x_S_2_ alloys with a uniform composition and a well-defined band gap^17^. Although very recently monolayer and multi-layer Mo_1−x_W_x_S_2_ alloys have been obtained by atomic layer deposition^12^, sulfurization of co-sputtered Mo_1−x_W_x_ thin films^37^ and other methods^38^,^39^, the monolithic Mo_1−x_W_x_S_2_ monolayers with tunable compositions and band gaps have not been realized by the conventional CVD method. In this study, we report a facile method for the synthesis of compositionally homogeneous monolayer Mo_1−x_W_x_S_2_ (x = 0–1) single crystals based on low-pressure (LP) CVD in a conventional CVD system. The band gaps of the synthesized monolayer alloys can be tailored by changing the Mo/W ratios, demonstrating the feasibility of realizing band gap engineering in 2D materials by the conventional CVD method.

## Results

### LP-CVD growth of monolayer Mo_1−x_W_x_S_2_ alloys

**Figure 1:**
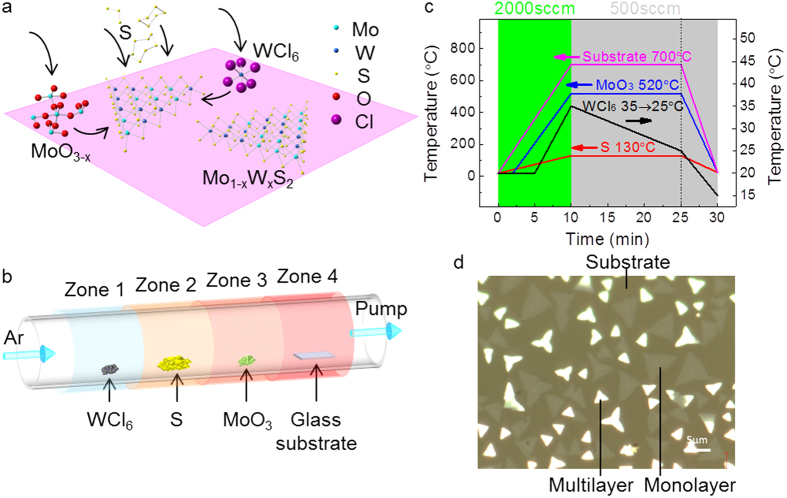
LP-CVD synthesis of monolayer Mo_1−x_W_x_S_2_ on glass substrates. (**a**) Schematic illustration of the surface reactions during CVD growth. (**b**) The arrangement of WCl_6_, sulfur, MoO_3_ and substrate in the CVD system. They were placed from upstream to downstream in four different furnace zones. The temperatures of each zone can be controlled independently. (**c**) Temperature profiles of the precursors and the substrate during CVD growth. The temperatures of each precursor and the substrate rose to their designed values within 10 min under 2000 sccm Ar flow rate. Then, the Ar flow was switched to 500 sccm for film growth. The temperature of the substrate, MoO_3_ and S sources were kept constant while the temperature of WCl_6_ gradually dropped from 35 to 25 °C in 15 min before cooling down. (**d**) Optical image showing the triangular flakes grew on the glass substrate (dark regions). Brighter ones are the multilayers and less bright ones are monolayers.

Different from previous CVD studies in which WO_3_ powders were used as the W source, in this study a highly volatile W-containing precursor WCl_6_ was chosen. WCl_6_ sublimates at much lower temperature below 100 °C^27^,^30^,^40^ and the temperature required for WS_2_ growth can be reduced to ~700 °C, which is very close to the CVD temperature of MoS_2_ using MoO_3_ as the precursor^30^. Therefore, MoO_3_ and WCl_6_ powders can be simultaneously used as the Mo and W sources for the CVD growth of monolayer Mo_1−x_W_x_S_2_ alloys and the Mo/W ratio can be tailored by tuning the MoO_3_ and WCl_6_ supplies. Figure 1a schematically illustrates the reactions during CVD process. Gas species MoO_3−x_ and WCl_6_ are transported to the glass substrate, adsorb to and diffuse on the surface before being completely sulfurized and rearranged to form a Mo_1−x_W_x_S_2_ alloy layer. Details about the CVD setup (Fig. 1b) and temperature profile (Fig. 1c) used in this study will be described in Methods section.

We noticed that the formation of MoS_2_/WS_2_ heterostructures cannot be completely avoided even if the WS_2_ growth temperature has been reduced to close to that of MoS_2_ by using the highly volatile WCl_6_ precursor. This is caused by uncontrollable sublimation of WCl_6_ and MoO_3_ during CVD temperature rising and cooling. The different temperature dependence of WCl_6_ and MoO_3_ sublimations leads to the separated deposition of MoS_2_ and WS_2_ in temperature rising/decreasing stages. To achieve homogeneous growth of monolithic Mo_1−x_W_x_S_2_ monolayers, the suppression of uncontrollable film deposition during temperature rising and cooling is critical. We utilized a high Ar flow rate to prevent the separate chemical deposition of MoS_2_ or WS_2_ during CVD temperature rising. It was found that the film deposition can be completely suppressed when the Ar flow rate is higher than ~2000 sccm (Fig. S1a,b, Supplementary Information). This suppression of deposition may originate from the low sublimation rates of sulfur source and MoO_3−x_ and WCl_6_ precursors at high pressures at which neither MoS_2 _nor WS_2_ can grow (Fig. S2, Supplementary Information). After the CVD temperature reaches to the designed value and is stabilized for 1 ~ 2 min, the Ar flow was then decreased to 500 sccm for homogeneous Mo_1−x_W_x_S_2_ growth.

It has been found that MoO_3_ suffers from the reduction by sulfur vapor and only partially reduced volatile MoO_3−x_ can be transported to substrates for Mo_1−x_W_x_S_2_ growth while nonvolatile MoS_2_ by excessive reduction forms a cap layer covering MoO_3_ source. The cap layer becomes thicker and thicker with CVD time. Since the production and transport of MoO_3−x_ require sulfur vapor and MoO_3−x_ vapor to diffuse through the MoS_2_ cap layer, the nonvolatile MoS_2_ cap layer results in the decrease of the MoO_3−x_ supply rate during CVD. In contrast, the simple sublimation of WCl_6_ keeps a constant supply rate at a designed CVD temperature. The de-coupling between the W and Mo supply rates leads to a compositional gradient in the grown monolayer Mo_1−x_W_x_S_2_ crystals with Mo-rich centers and W-rich rims (Fig. S3, Supplementary Information). Thus, to avoid the composition gradient, it is essential to maintain a constant ratio of Mo and W supply rates, which has been succeeded by gradually lowering down the heating temperature of WCl_6_ source during CVD growth as shown in Fig. 1c. Additionally, monolayer Mo_1−x_W_x_S_2_ samples with different compositions can be obtained by adjusting the amount of the WCl_6_ source.

### Microstructure characterization of Mo_1−x_W_x_S_2_ alloys

**Figure 2:**
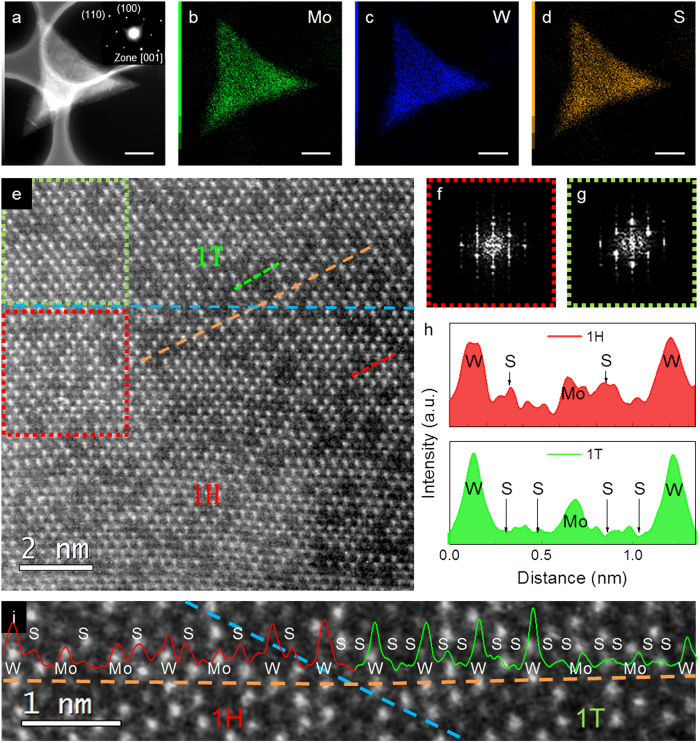
TEM characterization of structure and chemistry of a monolayer Mo_0.33_W_0.67_S_2_ alloy. (**a–d**) HAADF-STEM image and EDS element mappings of Mo, W and S taken from a triangular flake. The SAED pattern (the inset of (**a**)) reveals the single-crystal nature of the flake. The scale bar: 1 μm. (**e**) High resolution HAADF-STEM image showing the atomic structure and the coexistence of 1H and 1T phases. The phase boundary is marked by a blue dashed line. (**f,g**) Fourier transformed images from the rectangular regions in 1T (green dot line) and 1H (red dot line) phases in (**e)**. (**h**) Intensity profiles along the dashed lines in 1T and 1H regions. The upper red profile corresponds to the red dashed line in the 1H region and the lower green one is from the green dashed line in the 1T region. (**i**) Enlarged image of the vicinity of the orange dashed line across the phase boundary shown in (**e**). The intensity profile along the orange dashed line is overlaid on the image.

The LP-CVD method allows us to grow large-scale Mo_1−x_W_x_S_2_ films with tunable compositions and thickness. However, the continuous 2D films are difficult to be identified by optical microscopy and even scanning electron microscopy (SEM) (Fig. S9). In contrast, the as-grown Mo_1−x_W_x_S_2_ flakes are visible under an optical microscope (Fig. 1d), which are mainly used for the characterization of structure, chemistry and optical properties. The grey triangular flakes with the lateral size of around 5 μm are monolayer Mo_1−x_W_x_S_2_ and the bright ones with the size of 2 ~ 3 μm are multilayer ones, which can be elucidated by photoluminescence spectroscopy and high-angle annular dark field scanning transmission electron microscopy (HAADF-STEM) images (Fig. S4, Supplementary Information). The as-grown Mo_1−x_W_x_S_2_ flakes were transferred to holey carbon coated Cu grids for STEM characterization. As shown in Fig. 2a–d, the energy dispersive X-ray spectroscopy (EDS) mappings reveal the homogeneous distributions of Mo, W and S in a triangular flake, manifesting that the as-grown Mo_1−x_W_x_S_2_ crystals are a homogeneously mixed alloy, rather than a MoS_2_/WS_2_ heterostructure.

The atomic structure of the monolayer Mo_1−x_W_x_S_2_ alloys was visualized by HAADF-STEM (Fig. 2e) in which W atoms show a brighter contrast than Mo atoms because of the mass difference. Again, the random mixture of Mo and W atoms in the alloy is confirmed although the short-range chemical order and chemical fluctuation of both W and Mo can be identified in the atomic image. By simply counting the numbers of Mo and W, the compositions of the monolayer samples can be determined. For example, the composition of the sample shown in Fig. 2e is measured to be Mo_0.3_W_0.7_S_2_, which is consistent with the macroscopic composition of Mo_0.33_W_0.67_S_2_ determined by X-ray photoelectron spectroscopy (XPS) from the entire sample. The atomic images reveal that the as-grown Mo_1−x_W_x_S_2_ monolayer alloys contain two phases: metallic 1T and semiconductor 1H. Although the 1T phase has been observed in pristine MoS_2_ and WS_2_ before^41^,^42^,^43^,^44^,^45^,^46^,^47^,^48^,^49^,^50^,^51^, it is the first observation of 1T in the monolayer Mo_1−x_W_x_S_2_ grown by CVD. The phase identification is based on the contrast of S atoms. Since S atoms are much lighter than Mo and W, they are almost invisible in the metallic 1T phase due to the misaligned two sulfur layers as shown in Fig. 2e (upper part). In contrast, they are able to show the discriminable contrast in 1H in which two sulfur atomic layers project on each other in each sulfur atomic column and have enhanced intensity in the HAADF-STEM image (Fig. 2e, lower part). Hence, the difference between the atomic structure of 1H and 1T can be easily identified from the intensity profiles taken from the two phases (Fig. 2h). The interface between 1H and 1T phases is indicated by a blue dashed line in Fig. 2e. The intensity profile along the orange dashed line across the phase boundary is shown in Fig. 2i for detailed view. The phase boundary is formed by the displacement of one of the two S layers on a single side in agreement with previous observations in 1T/1H mixed monolayer MoS_2_^41^,^45^,^49^. The arrangement and orientation of Mo and W atoms remain undisturbed across the 1H/1T interface as confirmed by the nearly identical fast Fourier transform (FFT) patterns (Fig. 2e–g) from two phases (red short dashed line in 1H and green short dashed line in 1T region), similar to previous observations of the co-existence of 1T and 1H in monolayer MoS_2_^41^. Only 1T structure, rather than the distorted 1T (1T’) structure which is often observed in 1T-WS_2_ monolayer^47^,^52^, was observed even at locally W-rich compositions (x = 0.6 ~ 0.8). In addition, the domain sizes of the 1T phase appear larger than tens of nanometers since a single domain cannot be covered by the field of vision in STEM at a magnification large enough for the discrimination of 1T or 1H phase. The relation between the local Mo/W atomic ratio and 1T or 1H phase in the Mo_0.33_W_0.67_S_2_ 2D crystals is statistically analyzed by using the HAADF-STEM images randomly acquired from different regions of 1T and 1H phases. Both phases have a similar and homogeneous composition with small variation of local W proportion in the range from ~60% to ~80%. Based on a typical HAADF-STEM image, spatial distribution of W proportion x is shown in Fig. S5a,b (Supplementary Information), revealing a small compositional fluctuation of x = 0.58 ~ 0.78 in nanometer scale. Both the distribution of nearest Mo neighbors of Mo and Mo neighbors of W show similar Gaussian distribution centered near the macroscopic value (2.0 for x = 0.67) (Fig. S5c,d, Supplementary Information). The standard deviation of the average number of the nearest Mo neighbors of Mo and W atoms from 6(1−x) (x = 0.67 in this case) are 1.19 and 1.14 corresponding to Fig. S5c,d respectively, manifesting the atomic scale homogeneity of the as-grown sample.

### X-ray photoelectron spectroscopy (XPS) analysis

**Figure 3:**
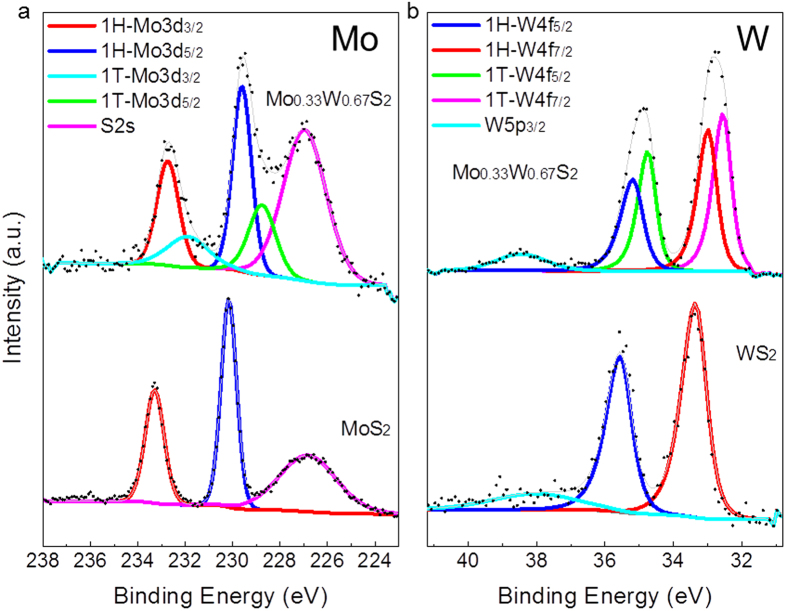
XPS measurements of the monolayer Mo_0.33_W_0.67_S_2_ alloy, monolithic MoS_2_ and WS_2_. (**a**) XPS spectra of Mo; and (**b**) XPS spectra of W. The co-existence of both 1H and 1T phase can be seen. Signals of 1H phase in Mo_0.33_W_0.67_S_2_ (upper **a**,**b**) show the binding energies close to the pristine (lower **a**) 1H-MoS_2_ and (lower **b**) 1H-WS_2_.

XPS was used to study the valence states and chemical composition of the 2D Mo_1−x_W_x_S_2_ alloys. Figure 3 (upper) shows the XPS results of Mo and W in the as-grown Mo_0.33_W_0.67_S_2_ alloy. For comparison, we also measured the XPS spectra of monolayer MoS_2_ (Fig. 3 lower left) and WS_2_ (Fig. 3 lower right) grown under the same CVD conditions by using single precursors. Based on the valence states, the trigonal prismatic 1H phase and octahedral 1T phase, can be identified from the XPS spectra^46^,^47^,^48^,^49^,^50^,^51^,^53^,^54^. In contrast to the spectra of pristine MoS_2_ and WS_2_ monolayers which only show the XPS peaks from the trigonal prismatic 1H phase, the splitting of Mo or W bands in Mo_1−x_W_x_S_2_ samples verifies the coexistence of 1H and 1T phases, confirming the HAADF-STEM observations. The core levels of Mo 3d_3/2_ at 232.8 eV and Mo 3d_5/2_ at 229.7 eV from the 1H phase in the Mo_0.33_W_0.67_S_2_ sample are ~0.3 eV lower than these binding energies of the pristine 1H MoS_2_. Mo 3d_3/2_ at 232.2 eV and Mo 3d_5/2_ at 229.1 eV from the 1T phase in Mo_0.33_W_0.67_S_2_ show ~0.6 eV deviation from the corresponding 1H signals in the sample. Likewise, W 4f_5/2_ (at 35.3 eV) and W 4f_7/2_ (at 33.1 eV) from the 1H phase in Mo_0.33_W_0.67_S_2_ are ~0.2 eV lower than those in the 1H WS_2_ while W 4f_5/2_ at 34.7 eV and W 4f_7/2_ at 32.5 eV from the 1T phase deviate for ~0.6 eV from the corresponding 1H signals in Mo_0.33_W_0.67_S_2_. According to the integral intensities of Mo and W peaks from each phase, the volume ratios of 1T to 1H phases and the compositions of each phase can be approximately estimated. By plotting the volume ratio of 1T phase against the W fraction, one can see that the fraction of 1T phase shows the maximum at the composition of about the half substitution of Mo atoms (Fig. S6). In this composition range, the 2D alloys have the maximum configurational entropy and the largest lattice strain caused by the atomic size difference between W and Mo although the size difference is very small. Both the chemical effect and local lattice strains are expected to reduce the energy barrier for the phase transformation from 1H to 1T. Moreover, the thermal strains induced by the mismatch of thermal expansion coefficients between Mo_1−x_W_x_S_2_ films and substrates may also play an important role in the formation of metastable 1T phase because the release of thermal strains by annealing can lead to the transformation from 1T to 1H. Since the 1T phase can transform into 1H phase with an unchanged Mo/W ratio by annealing at elevated temperatures, the 1H phase should be thermodynamically preferred one of the monolayer Mo_1−x_W_x_S_2_ crystals grown by CVD, which is similar to that in the pristine MoS_2_ and WS_2_^47^,^49^,^50^,^51^,^54^.

### Raman and photoluminescence spectroscopy of monolayer Mo_1−x_W_x_S_2_ crystals

**Figure 4:**
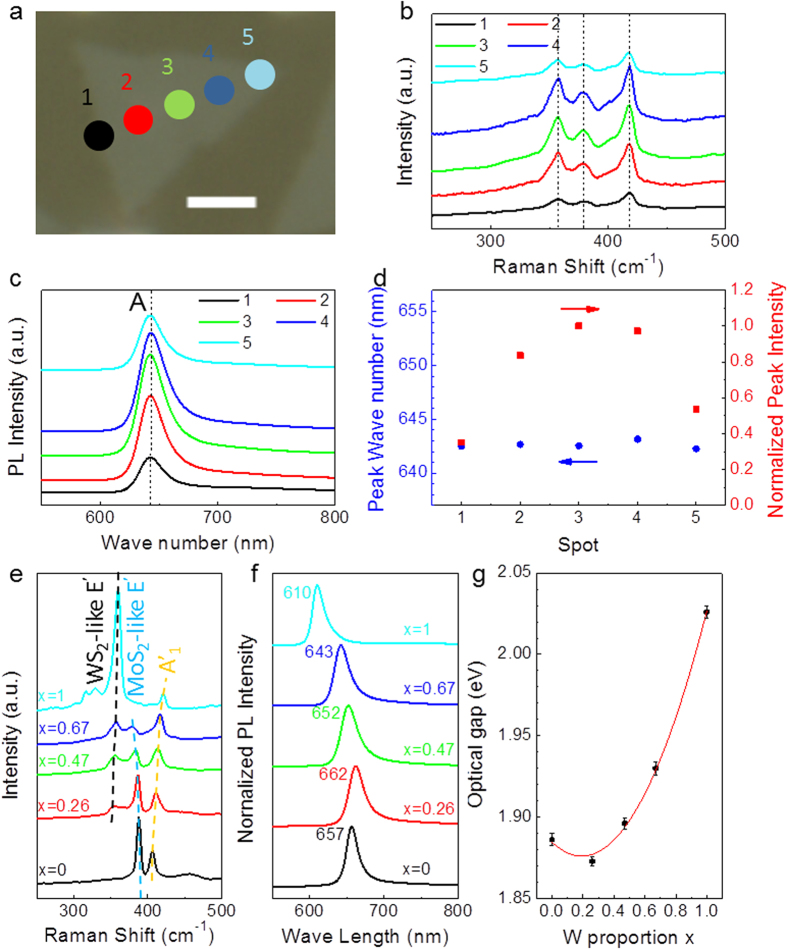
Raman and PL measurements of monolayer Mo_0.33_W_0.67_S_2_ (for **a–d**) and composition dependence of Raman and PL bands of monolayer Mo_1−x_W_x_S_2_ (for **e–g**). (**a**) Optical image of a triangular flake with marked five spots in a line cross the sample for Raman and PL measurements. Scale bar: 2 μm. (**b**) Raman spectra from the five spots. (**c**) PL spectra from the five spots. (**d**) Peak positions and intensities of the five PL spectra in (**c**). Both Raman and PL peaks show invisible changes in the positions. (**e,f**) The dependence of Raman and PL bands on Mo/W ratios. (**g**) The optical gap values as a function of W proportions in monolayer Mo_1−x_W_x_S_2_ alloys with error bars plotted by several measurements.

Raman spectroscope measurements were employed to characterize the structure of the monolayer Mo_1−x_W_x_S_2_ crystals. Figure 4b shows the Raman spectra acquired at five spots along a line across a monolayer Mo_0.33_W_0.67_S_2_ triangular flake on the glass substrate (Fig. 4a). The nearly identical spectra, in particular the positions of the characteristic Raman bands, further suggest the microscopic homogeneity of the as-grown 2D alloy crystals. The relatively lower intensities of the spectra from the spot 1 and 5 are because only a part of the laser beam shed on the sample at those locations. Three main phonon modes can be observed in the monolayer Mo_1−x_W_x_S_2_ alloys under 514.5 nm excitation, assigned to WS_2_-like E’ at 357.0 cm^−1^, MoS_2_-like E’ at 378.3 cm^−1^ and A’_1_ at 416.5 cm^−1^ (Fig. 4b). Here, Raman modes are noted in reference to the corresponding modes in monolayer MoS_2_ and WS_2_^55^. A’_1_ phonon mode is the out-of-plane vibration of sulfur atoms. For MoS_2_/WS_2_ heterostrutures, it splits into two frequencies corresponding to monolithic MoS_2_ and WS_2_^56^. The observed single A’_1_ mode without peak-splitting in this study provides Raman evidence that the Mo_1−x_W_x_S_2_ alloys grown by LP-CVD are the homogeneous mixture of Mo and W atoms in solid solution, not MoS_2_/WS_2_ heterostructures. In addition, weak Raman signals can also be seen in the low frequency range below 250 cm^−1^ (Fig. S7, Supplementary Information), which may be the signals from the 1T phase in the 2D alloys because similar Raman bands have been observed in 1T MoS_2_ and WS_2_ before^47^,^49^,^51^,^57^. Photoluminescence (PL) spectra from the five spots marked in Fig. 4a also show the near constant peak position of the *A* excitonic emission from the direct band gap at 643 nm (1.93 eV) (Fig. 4c,d), while the absence of the *B* excitonic emission signal, which is expected to appear at the lower wave length side of *A* exciton^58^,^59^, suggests the high crystallinity of the 2D crystal grown by LP-CVD^21^. The nearly identical *A* exciton peak positions verify the homogeneous exciton energy and therefore a consistent direct band gap across the monolayer Mo_1−x_W_x_S_2_ crystal.

## Discussion

Since the composition of the Mo_1−x_W_x_S_2_ monolayers can be customized by changing supply rates of Mo and W, Mo_1−x_W_x_S_2_ monolayers with various Mo/W ratio (x ranging from 0–1) were grown by simply changing the amount of MoO_3_ and WCl_6_ precursors (Table S1, Supplementary Information). The dependence of Raman and PL spectra on Mo/W ratios of the monolayer Mo_1−x_W_x_S_2_ alloys is shown in Fig. 4e,f. With the increase of W fraction, WS_2_-like E’ (+2LA) and A’_1_ Raman modes show blue-shift and MoS_2_-like E’ shows red-shift. Meanwhile, the relative intensity of WS_2_-like E’ (+2LA) increases and MoS_2_-like E’ decreases with the increase of W. From the PL results shown in Fig. 4f,g, the optical band gaps of the monolayer Mo_1−x_W_x_S_2_ can be calculated, which exhibits a bowing effect with the increase of Mo/W ratios. By fitting the curve with the following equation:


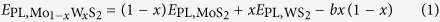


the bowing factor *b* was calculated to be ~0.26 eV, in good agreement with the reported value^12^. Therefore, the tunable direct band gaps of the monolayer Mo_1−x_W_x_S_2_ can be achieved by the LP-CVD method. Note that the full width at half maximum (FWHM) of the exciton peak in the PL spectra of the monolayer Mo_1−x_W_x_S_2_ alloys (69 meV for x = 0.26, 81 meV for x = 0.47, 80 meV for x = 0.67) is obviously larger than those of the pristine MoS_2_ (57 meV) and WS_2_ (60 meV) (Fig. 4f and Fig. S8, Supplementary Information). The enlargement of FWHM can be attributed to the large spread of band gaps under fixed x in the range of x = 0.33 ~ 0.60, which is the result of intrinsic polymorphism of the system predicted by a previous theoretical study^60^. It is worth noting that the formation of the 1T phase does not noticeably affect the PL peak positions since the peak shift cannot be seen in the annealed samples with nearly pure 1H phase.

The successful growth of the monolayer Mo_1−x_W_x_S_2_ alloys with homogeneously mixed W and Mo in 2D crystals benefits from the LP-CVD process that allows the simultaneous deposition of W and Mo at a constant atomic ratio. Moreover, the preference of hetero-atomic bonding under kinetic equilibrium conditions results in the random mixture of Mo and W atoms, which is maintained in Mo_1−x_W_x_S_2_ crystals without MoS_2_ and WS_2_ phase separation^13^. Therefore, as long as the ratio of supply rates of Mo- and W-containing precursors is adjusted to be constant during growth, chemically homogeneous Mo_1−x_W_x_S_2_ monolayers with tunable compositions and band gaps can be obtained by the LP-CVD method. After lowering down the deposition rate by reducing the amount of both precursors, growth of multilayers can be largely suppressed (Fig. S9a, Supplementary Information). And at the same time increasing deposition time leads to the coalescence of Mo_1−x_W_x_S_2_ monolayer flakes into a large area sheet (Fig. S9b, Supplementary Information). Our LP-CVD method for the growth of continuous Mo_1−x_W_x_S_2_ monolayer films shows notable flexibility in composition control and feasibility of mass production compared with conventional methods, such as chemical vapor transport (CVT) and mechanical/chemical exfoliation. Finally, the appearance of metastable 1T phase enables the incorporation of phase engineering into the alloying of TMDCs for device applications.

In summary, we have successfully developed a facile LP-CVD route for growing monolayer Mo_1−x_W_x_S_2_ crystals using a conventional CVD system. The monolayer Mo_1−x_W_x_S_2_ alloys with different compositions have intense *A* excitonic photoluminescence, which shows the strong dependence on Mo/W ratios of the monolayer alloys. As a result, the direct band gaps of the 2D Mo_1−x_W_x_S_2_ can be continuously tuned by altering W proportions in the 2D alloys. This work may pave a new way for fulfilling the applications of band gap engineering in 2D materials by LP-CVD growth of composition-controllable monolayer alloys. In addition, the co-existence of semiconducting 1H and metallic 1T phase is for the first time observed in CVD grown 2D dichalcogenides, which may be important in tuning electronic structures and exploring superconductivity in 2D alloy crystals^61^,^62^,^63^.

## Methods

### LP-CVD of monolayer Mo_1−x_W_x_S_2_ alloys

Three precursor sources and a substrate were placed in the sequence of WCl_6_ (Sigma Aldrich, purity 99.9%, ~5 mg), sulfur (Wako Pure Chemical Industry, purity 99%, 1.0 g), MoO_3_ (Sigma Aldrich, purity >99.5%, ~5 mg) and micro slide glass substrate (Matsunami Glass, S1214) from upstream to downstream in a 2-inch quartz tube as shown in Fig. 1b. Temperatures of the sources and substrate were controlled separately by heating in different furnace zones. Ultra-pure Argon was used as the carrier gas and the pressure inside the quartz tube was controlled by adjusting the Ar flow rate. As shown in the temperature profiles in Fig. 1c, the growth recipe involves three stages: temperature rising stage (first 10 min), growth stage (next 15 min) and cooling down stage (last 5 min). In the temperature rising stage, temperatures of the sources and the substrate rose to and were stabilized at their designed values (35 °C for WCl_6_, 130 °C for sulfur, 520 °C for MoO_3_ and 700 °C for substrate) in 10 min under the Ar flow rate of 2000 sccm (corresponding to 5.1 mbar). In the growth stage, Ar flow rate was switched to 500 sccm (2.1 mbar) to initiate the supply of all the species to substrate for film growth. The temperatures of the precursor sources of sulfur and MoO_3_ and the substrate were kept constant for 15 min while the temperature of WCl_6_ gradually dropped from 35 °C to 25 °C. Finally, in the cooling down stage, WCl_6_ was rapidly cooled down to 15 °C and the others to room temperature in 5 min to end the CVD growth. As-grown samples were transferred by using the standard PMMA-assisted transfer method. The as-grown samples were first spin-coated with poly(methyl methacrylate) (PMMA, Micro chem. 950K A4). 2M NaOH aq. was used to assist the detachment of samples from glass substrate. After fishing out the PMMA coated Mo_1−x_W_x_S_2_ samples by other substrates, PMMA was dissolved by acetone.

### Microstructure and property characterization

Raman and PL spectra were acquired by a confocal Raman spectroscopic system (Renishaw InVia RM 1000) with 514.5 nm excitation laser at the power of 10 mW and 2 mW respectively. The atomic structure and chemical analysis of the 2D alloys were characterized by HAADF-STEM and STEM-EDS by utilizing a high resolution TEM system (JEOL JEM-2100F) with double spherical aberration correctors for imaging and probing lenses. The TEM observations were performed at the acceleration voltage of 200 kV and the spatial resolution of the STEM is ~0.1 nm. XPS measurements were performed by AXIS ultra DLD (Shimazu) with Al Ka using X-ray monochromator. The peaks were fitted by Voigt functions with Lorentzian ratio of 30% and Gaussian ratio of 70% using the software CasaXPS from Casa software Ltd. The branching ratios for spin orbit split components of 3d and 4f orbitals are 3d_5/2_:3d_3/2_ = 3:2 and 4f_7/2_:4f_5/2_ = 4:3 respectively. Sensitive factors for Mo 3d and W 4f are 3.321 and 3.523 respectively.

## Additional Information

**How to cite this article**: Wang, Z. *et al.* Chemical Vapor Deposition of Monolayer Mo_1-x_W_x_S_2_ Crystals with Tunable Band Gaps. *Sci. Rep.* **6**, 21536; doi: 10.1038/srep21536 (2016).

## Supplementary Material

Supplementary Information

## References

[b1] Liu Z. *et al.* Strain and structure heterogeneity in MoS2 atomic layers grown by chemical vapour deposition. Nat. Commun. 5, 5246 (2014). 25404060 10.1038/ncomms6246

[b2] Wang Y. *et al.* Strain-induced direct–indirect bandgap transition and phonon modulation in monolayer WS2. Nano Res. 8, 2562–2572 (2015).

[b3] Conley H. J. *et al.* Bandgap engineering of strained monolayer and bilayer MoS2. Nano Lett. 13, 3626–3630 (2013). 23819588 10.1021/nl4014748

[b4] Castellanos-Gomez A. *et al.* Local strain engineering in atomically thin MoS2. Nano Lett. 13, 5361–5366 (2013). 24083520 10.1021/nl402875m

[b5] Scalise E., Houssa M., Pourtois G., Afanas’ev V. & Stesmans A. Strain-induced semiconductor to metal transition in the two-dimensional honeycomb structure of MoS2. Nano Res. 5, 43–48 (2012).

[b6] Pan J., Wang Z., Chen Q., Hu J. & Wang J. Band structure engineering of monolayer MoS2 by surface ligand functionalization for enhanced photoelectrochemical hydrogen production activity. Nanoscale 6, 13565–13571 (2014). 25268589 10.1039/c4nr02829e

[b7] Tang Q. & Jiang D. Stabilization and Band-Gap Tuning of the 1T-MoS2 Monolayer by Covalent Functionalization. Chem. Mater. 27, 3743–3748 (2015).

[b8] Mann J. *et al.* 2-Dimensional transition metal dichalcogenides with tunable direct band gaps: MoS2(1-x)Se2x monolayers. Adv. Mater. 26, 1399–1404 (2014). 24339159 10.1002/adma.201304389

[b9] Su S. H. *et al.* Band gap-tunable molybdenum sulfide selenide monolayer alloy. Small 10, 2589–2594 (2014). 24610642 10.1002/smll.201302893

[b10] Su S.-H. *et al.* Controllable Synthesis of Band-Gap-Tunable and Monolayer Transition-Metal Dichalcogenide Alloys. Front. Energy Res. 2, 1–8 (2014).

[b11] Ma Q. *et al.* Postgrowth tuning of the bandgap of single-layer molybdenum disulfide films by sulfur/selenium exchange. ACS Nano 8, 4672–4677 (2014). 24684434 10.1021/nn5004327

[b12] Song J.-G. *et al.* Controllable synthesis of molybdenum tungsten disulfide alloy for vertically composition-controlled multilayer. Nat. Commun. 6, 7817 (2015). 26204328 10.1038/ncomms8817 PMC4525162

[b13] Wei X.-L. *et al.* Modulating the atomic and electronic structures through alloying and heterostructure of single-layer MoS2. J. Mater. Chem. A 2, 2101–2109 (2014).

[b14] Li B. *et al.* Synthesis and Transport Properties of Large-Scale Alloy Co0.16Mo0.84S2 Bilayer Nanosheets. ACS Nano 9, 1257–1262 (2015). 25584859 10.1021/nn505048y

[b15] Chen Y. *et al.* Tunable band gap photoluminescence from atomically thin transition-metal dichalcogenide alloys. ACS Nano 7, 4610–4616 (2013). 23600688 10.1021/nn401420h

[b16] Zhang M. *et al.* Two-Dimensional Molybdenum Tungsten Diselenide Alloys: Photoluminescence, Raman Scattering, and Electrical Transport. ACS Nano 8, 7130–7137 (2014). 24884059 10.1021/nn5020566

[b17] Zheng S. *et al.* Monolayers of WxMo1−xS2 alloy heterostructure with in-plane composition variations. Appl. Phys. Lett. 106, 063113 (2015).

[b18] Xi J., Zhao T., Wang D. & Shuai Z. Tunable electronic properties of two-dimensional transition metal dichalcogenide alloys: A first-principles prediction. J. Phys. Chem. Lett. 5, 285–291 (2014). 26270701 10.1021/jz402375s

[b19] Dumcenco D. O., Kobayashi H., Liu Z., Huang Y.-S. & Suenaga K. Visualization and quantification of transition metal atomic mixing in Mo1-xWxS2 single layers. Nat. Commun. 4, 1351 (2013). 23322039 10.1038/ncomms2351 PMC3564975

[b20] Lee Y.-H. *et al.* Synthesis of large-area MoS2 atomic layers with chemical vapor deposition. Adv. Mater. 24, 2320–2325 (2012). 22467187 10.1002/adma.201104798

[b21] Ji Q. *et al.* Epitaxial monolayer MoS2 on mica with novel photoluminescence. Nano Lett. 13, 3870–3877 (2013). 23899342 10.1021/nl401938t

[b22] Lee Y.-H. *et al.* Synthesis and transfer of single-layer transition metal disulfides on diverse surfaces. Nano Lett. 13, 1852–1857 (2013). 23506011 10.1021/nl400687n

[b23] Najmaei S. *et al.* Vapour phase growth and grain boundary structure of molybdenum disulphide atomic layers. Nat. Mater. 12, 754–759 (2013). 23749265 10.1038/nmat3673

[b24] Shi J. *et al.* Controllable Growth and Transfer of Monolayer MoS2 on Au Foils and Its Potential Application In Hydrogen Evolution Reaction. ACS Nano 8, 10196–10204 (2014). 25211696 10.1021/nn503211t

[b25] Ji Q. *et al.* Unravelling Orientation Distribution and Merging Behavior of Monolayer MoS2 Domains on Sapphire. Nano Lett. 15, 198–205 (2015). 25434826 10.1021/nl503373x

[b26] Zhang Y. *et al.* Controlled Growth of High-Quality Monolayer WS2 Layers on Sapphire. ACS Nano 7, 8963–8971 (2013). 24047054 10.1021/nn403454e

[b27] Okada M. *et al.* Direct Chemical Vapor Deposition Growth of WS2 Atomic Layers on Hexagonal Boron Nitride. ACS Nano 8, 8273–8277 (2014). 25093606 10.1021/nn503093k

[b28] Rong Y. *et al.* Controlling sulphur precursor addition for large single crystal domains of WS2. Nanoscale 6, 12096–12103 (2014). 25195869 10.1039/c4nr04091k

[b29] Cong C. *et al.* Synthesis and optical properties of large-area single-crystalline 2D semiconductor WS2 monolayer from chemical vapor deposition. Adv. Opt. Mater. 2, 131–136 (2014).

[b30] Park J. *et al.* Layer-modulated synthesis of uniform tungsten disulfide nanosheet using gas-phase precursors. Nanoscale 7, 1308–1313 (2015). 25361429 10.1039/c4nr04292a

[b31] van der Zande A. M. *et al.* Grains and grain boundaries in highly crystalline monolayer molybdenum disulphide. Nat. Mater. 12, 554–61 (2013). 23644523 10.1038/nmat3633

[b32] Ling X. *et al.* Role of the Seeding Promoter in MoS2 Growth by Chemical Vapor Deposition. Nano Lett. 14, 464–472 (2014). 24475747 10.1021/nl4033704

[b33] Fu Q. *et al.* Controllable synthesis of high quality monolayer WS2 on a SiO2/Si substrate by chemical vapor deposition. RSC Adv. 2, 15795–15799 (2015).

[b34] Xu Z. *et al.* Synthesis and Transfer of Large-Area Monolayer WS2 Crystals: Moving Toward the Recyclable Use of Sapphire. ACS Nano 9, 6178–6187 (2015). 25961515 10.1021/acsnano.5b01480

[b35] Dumcenco D. *et al.* Large-area Epitaxial Monolayer MoS2. ACS Nano 9, 4611–4620 (2015). 25843548 10.1021/acsnano.5b01281 PMC4415455

[b36] Tan Y. *et al.* Monolayer MoS2 Films Supported by 3D Nanoporous Metals for High-Efficiency Electrocatalytic Hydrogen Production. Adv. Mater. 26, 8023–8028 (2014). 25363090 10.1002/adma.201403808

[b37] Liu H., Antwi K. K. A., Chua S. & Chi D. Vapor-phase growth and characterization of Mo(1-x)W(x)S2 (0 ≤ x ≤ 1) atomic layers on 2-inch sapphire substrates. Nanoscale 6, 624–629 (2014). 24253383 10.1039/c3nr04515c

[b38] George A. S. *et al.* Wafer Scale Synthesis and High Resolution Structural Characterization of Atomically Thin MoS2 Layers. Adv. Funct. Mater. 24, 7461–7466 (2014).

[b39] Lin Z. *et al.* Facile synthesis of MoS2 and MoxW1-xS2 triangular monolayers. APL Mater. 2, 092804 (2014).

[b40] Yang Z. *et al.* Realization of high Curie temperature ferromagnetism in atomically thin MoS2 and WS2 nanosheets with uniform and flower-like morphology. Nanoscale 7, 650–658 (2015). 25427772 10.1039/c4nr06141a

[b41] Eda G. *et al.* Coherent atomic and electronic heterostructures of single-layer MoS2. ACS Nano 6, 7311–7317 (2012). 22799455 10.1021/nn302422x

[b42] Gao G. *et al.* Charge Mediated Semiconducting-to-Metallic Phase Transition in Molybdenum Disulfide Monolayer and Hydrogen Evolution Reaction in New 1T′ Phase. J. Phys. Chem. C 119, 13124–13128 (2015).

[b43] Kang Y. *et al.* Plasmonic hot electron induced structural phase transition in a MoS2 monolayer. Adv. Mater. 26, 6467–6471 (2014). 25100132 10.1002/adma.201401802

[b44] Duerloo K.-A. N., Li Y. & Reed E. J. Structural phase transitions in two-dimensional Mo- and W-dichalcogenide monolayers. Nat. Commun. 5, 4214 (2014). 24981779 10.1038/ncomms5214

[b45] Lin Y., Dumcenco D. O., Huang Y. & Suenaga K. Atomic mechanism of the semiconducting-to- metallic phase transition in single-layered MoS2. Nat. Nanotechnol. 9, 391–396 (2014). 24747841 10.1038/nnano.2014.64

[b46] Voiry D. *et al.* Conducting MoS2 nanosheets as catalysts for hydrogen evolution reaction. Nano Lett. 13, 6222–6227 (2013). 24251828 10.1021/nl403661s

[b47] Voiry D. *et al.* Enhanced catalytic activity in strained chemically exfoliated WS2 nanosheets for hydrogen evolution. Nat. Mater. 12, 850–855 (2013). 23832127 10.1038/nmat3700

[b48] Acerce M., Voiry D. & Chhowalla M. Metallic 1T phase MoS2 nanosheets as supercapacitor electrode materials. Nat. Nanotechnol. 10, 313–318 (2015). 25799518 10.1038/nnano.2015.40

[b49] Kappera R. *et al.* Phase-engineered low-resistance contacts for ultrathin MoS2 transistors. Nat. Mater. 13, 1128–1134 (2014). 25173581 10.1038/nmat4080

[b50] Eda G. *et al.* Photoluminescence from chemically exfoliated MoS2. Nano Lett. 11, 5111–5116 (2011). 22035145 10.1021/nl201874w

[b51] Guo Y. *et al.* Probing the Dynamics of the Metallic-to-Semiconducting Structural Phase Transformation in MoS2 Crystals. Nano Lett. 15, 5081–5088 (2015). 26134736 10.1021/acs.nanolett.5b01196

[b52] Mahler B., Hoepfner V., Liao K. & Ozin G. Colloidal Synthesis of 1T-WS 2 and 2H-WS 2 Nanosheets: Ap- plications for Photocatalytic Hydrogen Evolution. J. Am. Chem. Soc. 136, 14121–14127 (2014). 25220034 10.1021/ja506261t

[b53] Chou S. S. *et al.* Controlling the Metal to Semiconductor Transition of MoS2 and WS2 in Solution. J. Am. Chem. Soc. 137, 1742–1745 (2015). 25608577 10.1021/ja5107145

[b54] Yan S. *et al.* Enhancement of magnetism by structural phase transition in MoS2. Appl. Phys. Lett. 106, 012408 (2015).

[b55] Terrones H. *et al.* New First Order Raman-active Modes in Few Layered Transition Metal Dichalcogenides. Sci. Rep. 4, 4215 (2014). 24572993 10.1038/srep04215 PMC5379439

[b56] Gong Y. *et al.* Vertical and in-plane heterostructures from WS2/MoS2 monolayers. Nat. Mater. 13, 1135–1142 (2014). 25262094 10.1038/nmat4091

[b57] Liu Q. *et al.* Stable Metallic 1T-WS2 Nanoribbons Intercalated with Ammonia Ions: The Correlation between Structure and Electrical/Optical Properties. Adv. Mater. 27, 4837–4844 (2015). 26177725 10.1002/adma.201502134

[b58] Yu Y. *et al.* Controlled scalable synthesis of uniform, high-quality monolayer and few-layer MoS2 films. Sci. Rep. 3, 1866 (2013). 23689610 10.1038/srep01866 PMC3659320

[b59] Wang X., Feng H., Wu Y. & Jiao L. Controlled Synthesis of Highly Crystalline MoS2 Flakes by Chemical Vapor Deposition. J. Am. Chem. Soc. 135, 5304–5307 (2013). 23489053 10.1021/ja4013485

[b60] Kutana A., Penev E. S. & Yakobson B. I. Engineering electronic properties of layered transition-metal dichalcogenide compounds through alloying. Nanoscale 6, 5820–5825 (2014). 24744083 10.1039/c4nr00177j

[b61] Van Maaren M. H., Harland H. B. & Havinga E. E. Critical carrier concentration for superconductivity in a CuRh2Se4 - based system. Phys. Lett. A 30, 204–205 (1969).

[b62] Zakharov O., Cohen M. L., Louie S. G. & Penn D. R. Dynamical screening at the metal - semiconductor interface and excitonic superconductivity. J. Phys. Condens. Matter 9, 8501–8514 (1999).

[b63] Ting C. S., Talwar D. N. & Ngai K. L. Possible Mechanism of Superconductivity in Metal-Semiconductor Eutectic Alloys. Phys. Rev. Lett 45, 1213–1216 (1980).

