# Supplementary material for: Chemical Vapor Deposition of Monolayer Mo1−xWxS2 Crystals with Tunable Band Gaps
Source: Sci Rep. 2016 Feb 22;6:21536. doi: 10.1038/srep21536 (PMC4761910; doi:10.1038/srep21536)
Supplement: Supplementary Information [file srep21536-s1.pdf]

Supplementary Information for

Chemical Vapor Deposition of Monolayer $Mo_{1-x}W_xS_2$ Crystals with Tunable Band Gaps

Ziqian Wang[1], *Pan Liu*[2], *Yoshikazu Ito*[2,3], *Shoucong Ning*[4], *Yongwen Tan*[2], *Takeshi Fujita*[2,3], *Akihiko Hirata*[2,3], *Mingwei Chen*[1,2,3,]*

[1]Department of Materials Science, Graduate School of Engineering, Tohoku University, Sendai 980-8577, Japan

[2]WPI Advanced Institute for Materials Research, Tohoku University, Sendai 980-8577, Japan

[3]CREST, JST, 4-1-8 Honcho Kawaguchi, Saitama 332-0012, Japan

[4]Department of Mechanical and Aerospace Engineering, School of Engineering, Hong Kong University of Science and Technology, Clear Water Bay, Kowloon, Hong Kong SAR

*Address correspondence to: mwchen@wpi-aimr.tohoku.ac.jp

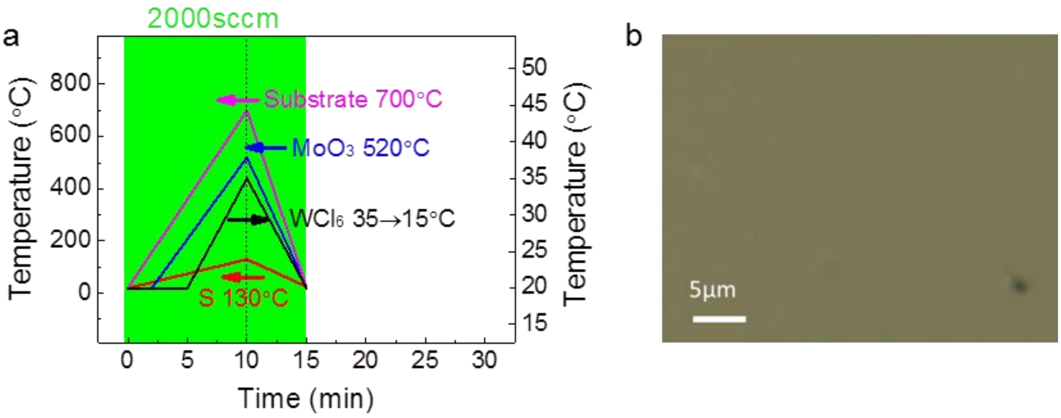

Figure S1. (a) The temperature profiles of the precursors and substrate during temperature rising and cooling at the Ar flow rate of 2000 sccm. All the reactants were cooled down right after temperature rose to the designed values in 10 min. (b) Optical image of the glass substrate after temperature rising and cooling. No trace of film growth can be observed from the substrate.

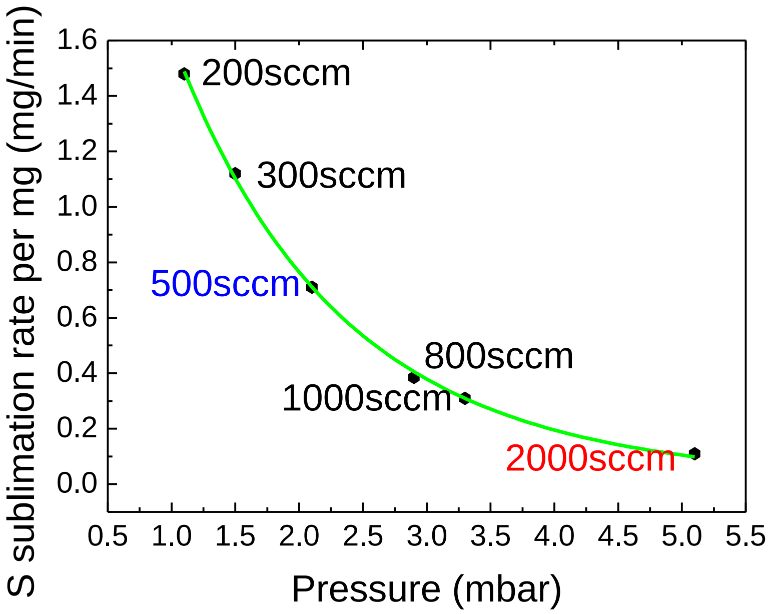

Figure S2. The dependence of sulfur sublimation rate on Ar flow rate (or pressure) at a constant heating temperature of 113 ℃. The sulfur sublimation rate decreases significantly with increasing Ar flow rate (or increasing pressure). 500 sccm (2.1 mbar) was used for deposition and 2000 sccm (5.1 mbar) was used to suppress deposition.

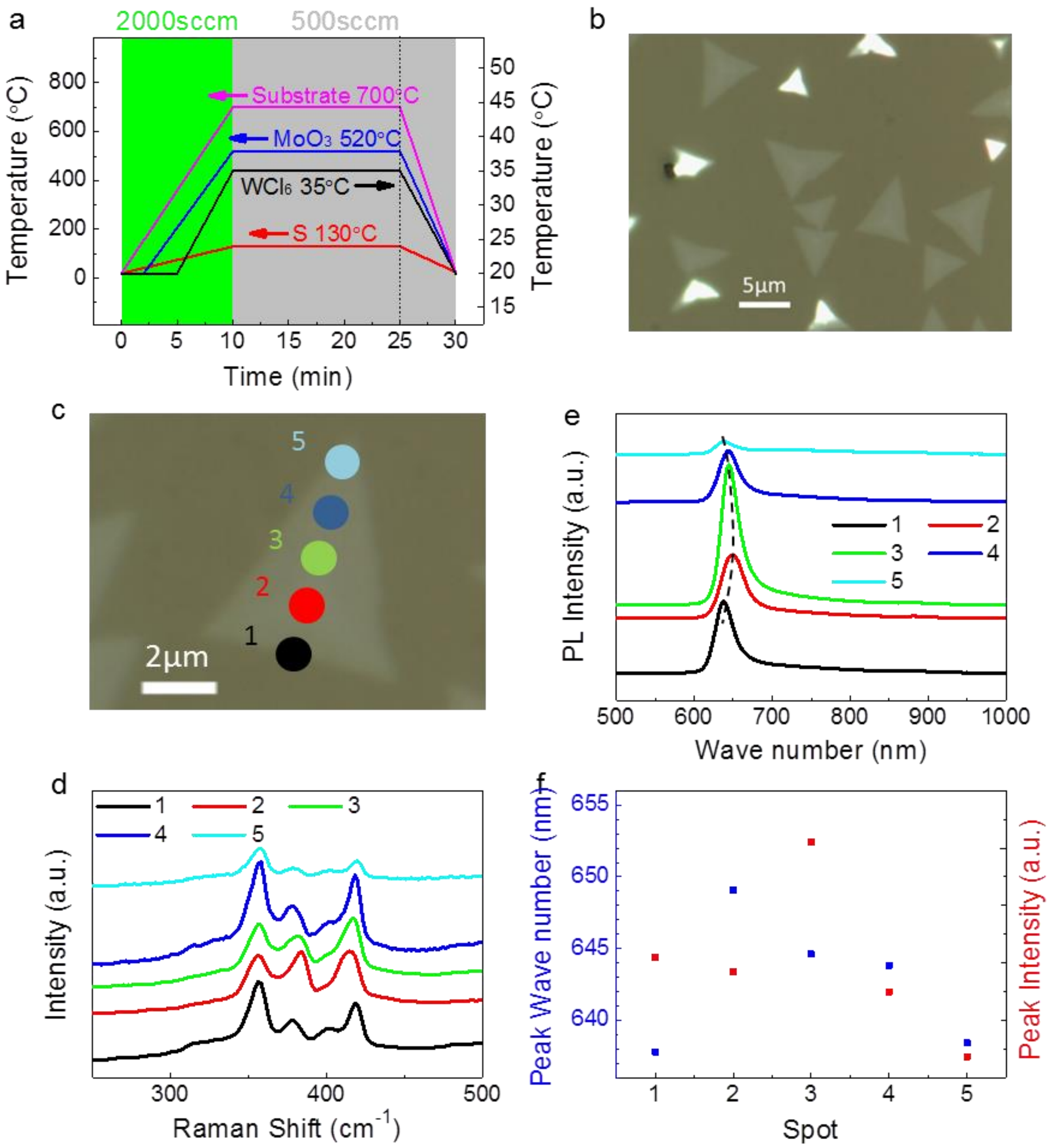

Figure S3. Monolayer $Mo_{1-x}W_xS_2$ single crystals grown at a constant temperature of the WCl_6 source. (a) Temperature profiles of the precursors and substrate. During temperature rising and cooling, the Ar flow rate is 2000 sccm and kept constant at 500 sccm for deposition. (b) Optical image showing single-crystal flakes. Brighter triangles are multilayers and less bright ones are monolayers. (c) Five spots in a line across a flake for Raman and PL measurements. (d, e) Raman and PL spectra taken from the five spots in (c). (f) PL peak positions and intensities extracted from (e). Raman and PL results indicate that a compositional gradient existed in the sample with Mo-rich in the center and W-rich in the rim.

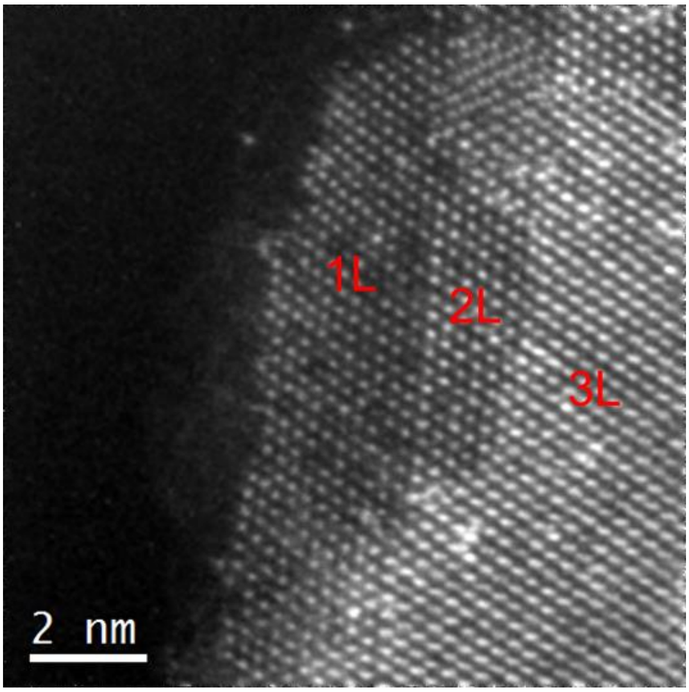

Figure S4. HAADF-STEM image taken from the edge of a multilayer $Mo_{0.33}W_{0.67}S_2$ sample. Monolayer (1L), bilayer (2L) and tri-layer (3L) regions can be identified from the contrast difference. Mo and W atoms can hardly be discriminated in 2L and 3L regions because of the overlapping.

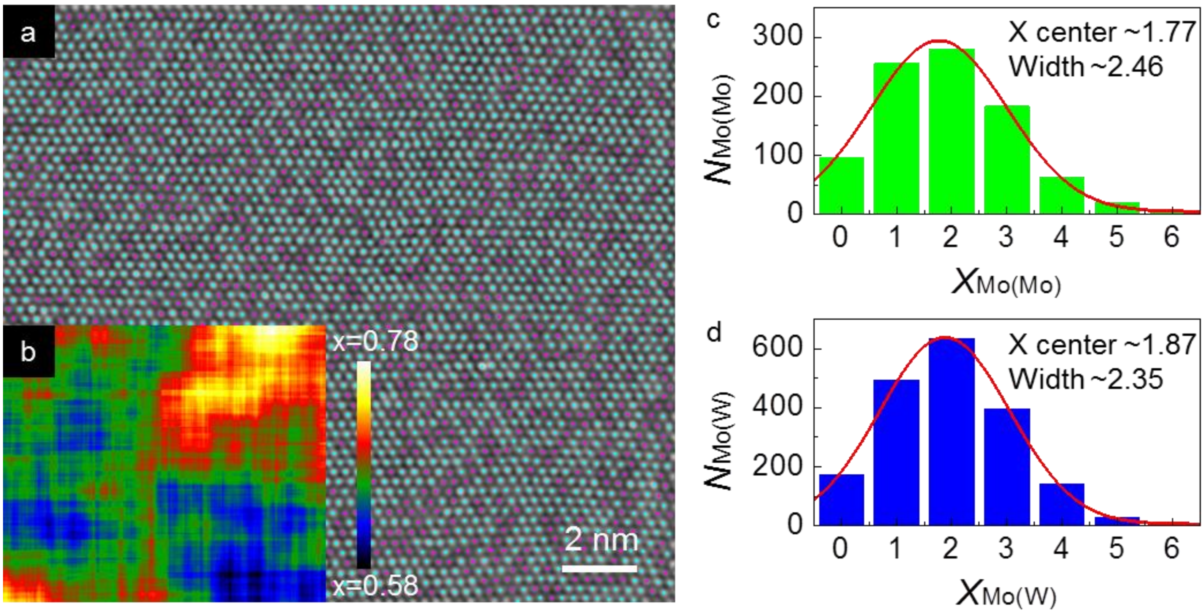

Figure S5. Spatial distribution of local W proportion x calculated from a HAADF-STEM image of $Mo_{1-x}W_xS_2$ monolayer. (a) Mo/W atoms are recognized from a HAADF-STEM image: W as cyan points and Mo as purple points. (b) Local W proportion x mapping of the same image. Local W proportion within the range of x=0.58~0.78 throughout the image indicates good spatial homogeneity in nanometer scale. (c, d) Distributions of nearest Mo neighbors of Mo and W calculated from Figure (a). Herein, $X_{A(B)}$ means the number of nearest A neighbors of a B atom and $N_{A(B)}$ means the number of B atoms with a certain nearest A neighbor number. Both the distribution of Mo neighbors of Mo and Mo neighbors of W show similar Gaussian distribution centered near the macroscopic value (2.0 for $Mo_{0.33}W_{0.67}S_2$).

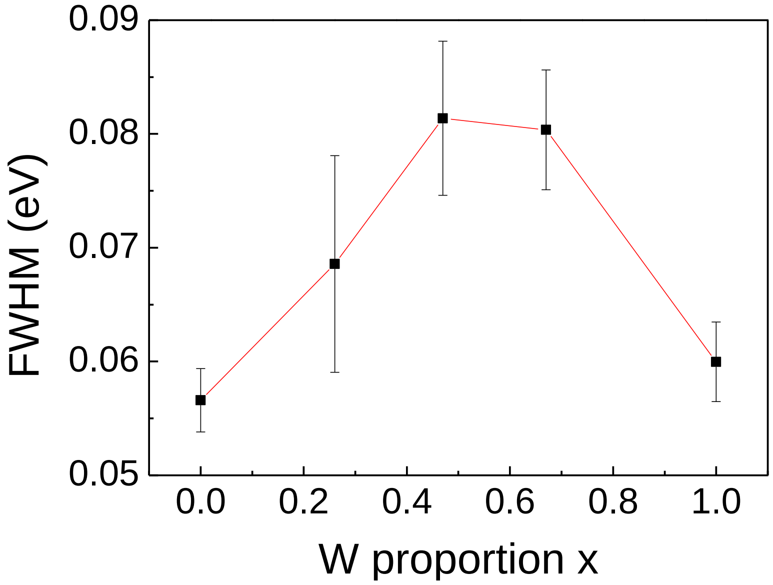

Figure S6. Relation between 1T phase ratio and W fraction in the as-grown $Mo_{1-x}W_xS_2$ samples. The as-grown samples were obtained by the same growth recipe except that different amounts of WCl_6 powder were used to adjust the composition of $Mo_{1-x}W_xS_2$. The 1T phase ratio increase to the maximum as the composition approached half substitution of Mo atoms by W atoms.

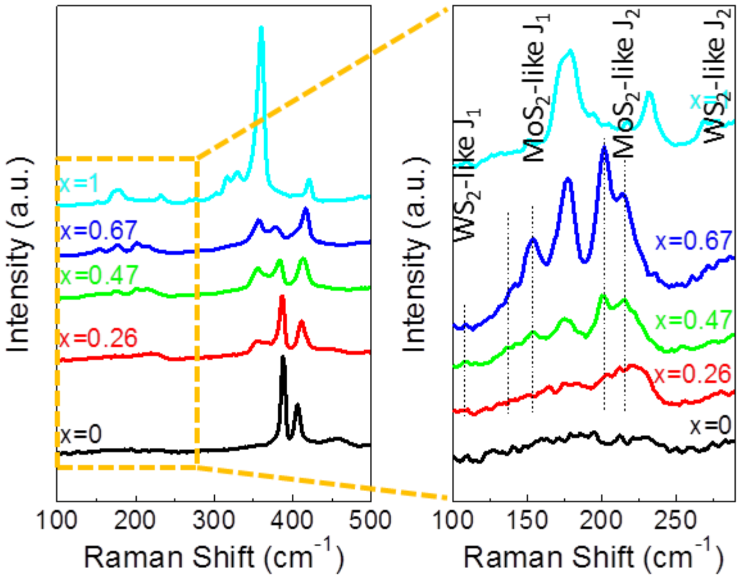

Figure S7. Low-frequency Raman spectra of monolayer $Mo_{1-x}W_xS_2$ with different composition. Weak peaks locating in the low frequency range from 100 cm^{-1} to 300 cm^{-1} are considered to be the signals from 1T-$Mo_{1-x}W_xS_2$, since previous reports on 1T-MoS_2 and 1T-WS_2 show the similar Raman signals in the same range.[R1-R4] The peaks appeared at ~130cm^{-1} and ~200cm^{-1} cannot be attributed to any reported ones and are probably the feature 1T signals of the alloyed $Mo_{1-x}W_xS_2$.

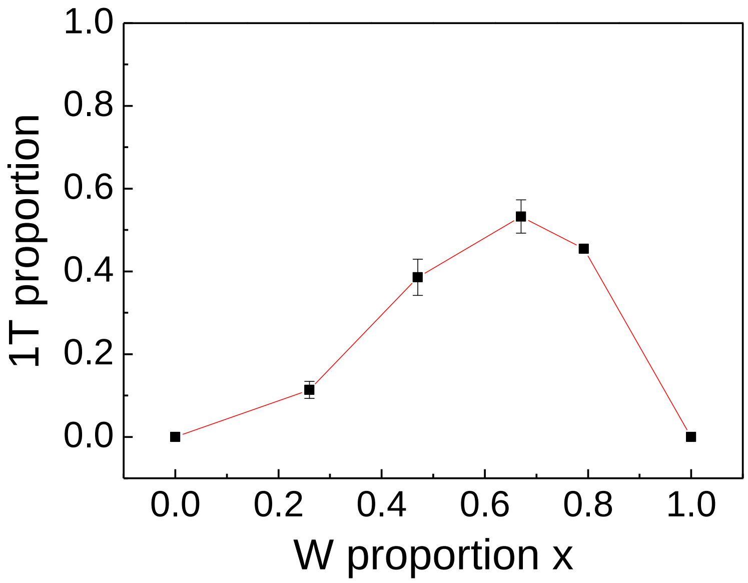

Figure S8. Composition dependence of the FWHM of PL of as-grown $Mo_{1-x}W_xS_2$ monolayers. Error bars are statistically calculated from the standard deviation of over five data points at each composition.

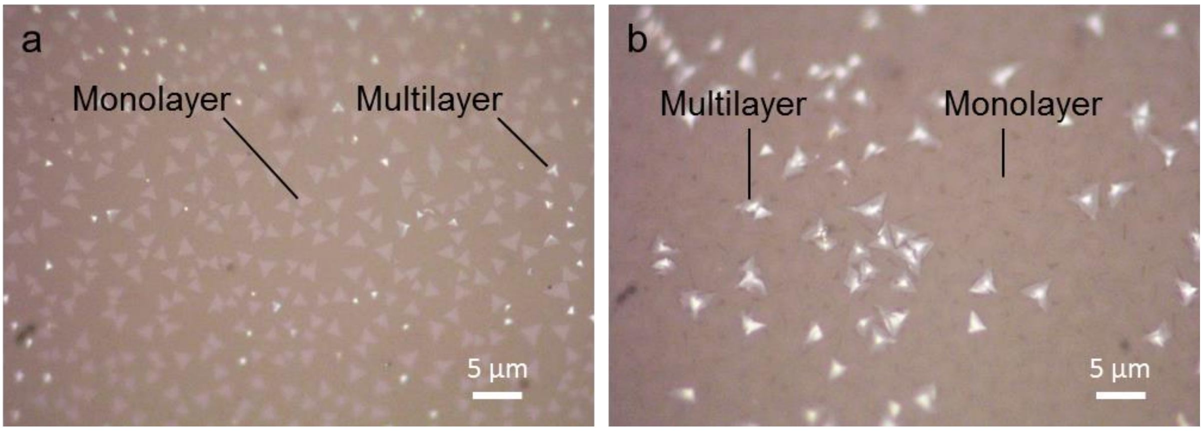

Figure S9. Optical images of as-grown $Mo_{1-x}W_xS_2$ after the control of deposition rate and growth time. (a) The formation of multilayer flakes (bright tiny triangles) can be suppressed by lowering down the deposition rate (reducing the amount of both WCl_6 and MoO_3 precursors). (b) Large-area continuous monolayer sheets can be obtained by controlling deposition rate and further increasing deposition time.

Table S1. Different amounts of MoO_3 and WCl_6 precursors used for the growth of $Mo_{1-x}W_xS_2$ monolayers with various Mo/W ratios.

x	MoO_3 (mg)	WCl_6 (mg)
0	10.0	0
0.26	5.0	2.0
0.47	5.0	3.5
0.67	5.0	5.0
1	0	10.0

Cited Reference in the Caption of Figure S7

[R1] D. Voiry, H. Yamaguchi, J. Li, R. Silva, D. C. B. Alves, T. Fujita, M. Chen, T. Asefa, V. B. Shenoy, G. Eda, M. Chhowalla, *Nat. Mater.* **2013**, *12*, 850.

[R2] R. Kappera, D. Voiry, S. E. Yalcin, B. Branch, G. Gupta, A. D. Mohite, M. Chhowalla, *Nat. Mater.* **2014**, *13*, 1128.

[R3] Y. Guo, D. Sun, B. Ouyang, A. Raja, J. Song, T. F. Heinz, L. E. Brus, *Nano Lett.* **2015**, *15*, 5081−5088.

[R4] Q. Liu, X. Li, Z. Xiao, Y. Zhou, H. Chen, A. Khalil, T. Xiang, J. Xu, W. Chu, X. Wu, J. Yang, C. Wang, Y. Xiong, C. Jin, P. M. Ajayan, L. Song, Adv. Mater. **2015**, 27, 4837.
